# A scoping review of online international student collaboration in occupational therapy education

**DOI:** 10.1177/03080226221086221

**Published:** 2022-06-11

**Authors:** Sinéad M. Hynes, Caroline Hills, Kristina Orban

**Affiliations:** 1 Discipline of Occupational Therapy, School of Health Sciences, 8799 National University of Ireland Galway, Ireland; 2 Occupational Therapy and Occupational Science, Department of Health Sciences, Faculty of Medicine, 59568 Lund University, Sweden

**Keywords:** Online collaboration, occupational therapy student, internationalisation, curriculum design, education, scoping review

## Abstract

**Introduction:**

For occupational therapy students, international experiences and access to a global curriculum develops understanding of broad cultural and contextual determinants of health and wellbeing. International placements or study abroad opportunities are not possible for many students and many universities are developing alternative internationalisation opportunities. The aim of this review was to determine what is known from the existing literature on the use of online international student collaboration in occupational therapy curricula.

**Method:**

A scoping review design was used to search relevant literature on online international student collaboration in occupational therapy education, following a methodological framework for conducting scoping reviews. Seven databases were searched. Search included all articles published up until November 2020.

**Findings:**

The database searches yielded a total of 2011 results. Following screening and review of articles ten papers met the inclusion criteria and were included in the review. The studies were charted and discussed in the areas of format of the online interactions, reported outcomes of the online interactions, barriers and facilitators in implementation.

**Conclusion:**

Findings inform curricula designers establishing online international learning and those conducting research in this area. Outcomes indicate the breadth of student learning including culture, diversity, as well as the social determinants of occupational engagement and participation.

**Registration:**

Protocol Registered: 2020-07-06, available on Open Science Framework (OSF) at https://osf.io/wfkjy

## Introduction

The World Federation of Occupational Therapists Minimum standards for the education of occupational therapists delineate the requirement for a global perspective in occupationally focussed curricula. Programs must aim for students to be able to work with diverse populations, as the profession needs to be anticipatory rather than just responsive to global health challenges. This includes an understanding of what influences health and wellbeing at a micro and meso (institutional) and macro level (WFOT, 2016) so that students develop an awareness of the contextual determinants of health and wellbeing. Students therefore need to appreciate diversity and develop the ability to identify occupational injustices so as to become future agents of social change for individuals, communities and populations. Frank (2012) argued internationalisation is essential in occupational therapy programs as this enables the exploration of concepts of globalisation through evaluation of occupational science in the international context. To ‘set the context for the transnational advocacy networks now operating that link occupational therapy with occupational sciences in service for a shared moral philosophy of social hope’ (Frank, 2012: p. 25). Globalisation is the interdependence, integration and interconnectedness of the world that has been influenced by the World Wide Web and a networked society (Frank, 2012). There is evidence of globalisation in occupational therapy curricula through international learning activities including international practice placements (Davies, 2017) and international interuniversity blended mobility (online and face-to-face) student exchanges (Truman et al., 2021) and interuniversity online learning (Aldrich, 2015; Psychouli et al., 2020).

Virtual learning has been used, in various modalities, in third level institutions for over a decade but has been catapulted into being the primary teaching modality for many over the past 2 years due to the COVID-19 pandemic (Langegard et al., 2021). The benefit for students of online learning has always been the flexibility of access; enabling asynchronous learning, helping education fit with learners’ other life commitments, reduced time in travel making courses more accessible and the use of Web 2.0 technologies. Web 2.0 technologies are those that facilitate sharing, collaboration and networking, for example, podcasting, social networking and communities of practice (Hills et al., 2016). The barriers to online and virtual learning include access to computers, reliable internet connection, students’ skills in the online technologies, student isolation and an inappropriate study environment, with many students working in their bedrooms (Hills et al., 2016; Langegard et al., 2021). Nevertheless, third level institutions had speedily transitioned from traditional classroom activities to the virtual environment to maintain course delivery during the COVID-19 pandemic. Before the pandemic however, Web 2.0 technologies had opened the door for wider collaboration including interuniversity, interprofessional and international learning.

Online international collaborations (sometimes called internationalisation at home or synchronous online education or cultural exchange at home) refer to students from two or more international (occupational therapy in this case) programmes coming together online to work towards shared learning objectives (Zadnik et al., 2019). The technology involved can vary but usually include meetings via a video conference platform of some kind [e.g. secure platforms such as Microsoft Teams (Microsoft, 2021) or Zoom platforms (Zoom Video Communications Inc, 2021)] and the use of social media collaborative learning spaces (e.g. Facebook closed groups and WhatsApp). Email, to develop discussions in a more informal nonsynchronous way, may also be included outside of conference meetings. These modalities enable students to participate in an international discourse. This process, when enabled by a skilled facilitator, allows students to gain insight into different cultures as well as an understanding of the scope of occupational therapy practice in different societal contexts. There are however challenges with international collaborations these include, language, time difference for synchronous and asynchronous learning, with some students reporting these make course work time consuming (Cabatan and Grajo, 2017; Psychouli et al., 2020). Despite these challenges, online international learning is being used to achieve learning outcomes in internationalisation and other areas of curricula. No review exists on the use of online international student collaboration in occupational therapy training and education to inform those planning to develop such meaningful digital learning spaces. The review will focus on the following question: What is known from the existing literature on the use of online international student collaboration in occupational therapy curricula?

## Methodology

The review protocol was registered: 2020-07-06, following a search to ensure no other reviews or protocols were published in the area (available on Open Science Framework (OSF) at https://osf.io/wfkjy). Given the broad nature of the research topic, a scoping review methodology (Arksey and O'Malley, 2005) was used. The framework by Arksey & O’Malley’s (2005) for conducting scoping reviews was followed but included amendments where appropriate as recommended by Levac et al. (2010) and Peters et al. (2020). We have followed best practice reporting criteria outlined in PRISMA-ScR (Tricco et al., 2018) at the end of this review (Table 1).

**Table 1.** Preferred Reporting Items for Systematic Reviews and Meta-Analyses extension for Scoping Reviews (PRISMA-ScR) checklist.

Section	Item	PRISMA-ScR checklist item	Reported on Page #
Title			
Title	1	Identify the report as a scoping review	1
Abstract			
Structured summary	2	Provide a structured summary that includes (as applicable): Background, objectives, eligibility criteria, sources of evidence, charting methods, results and conclusions that relate to the review questions and objectives	1
Introduction			
Rationale	3	Describe the rationale for the review in the context of what is already known. Explain why the review questions/objectives lend themselves to a scoping review approach	2
Objectives	4	Provide an explicit statement of the questions and objectives being addressed with reference to their key elements (e.g. population or participants, concepts and context) or other relevant key elements used to conceptualise the review questions and/or objectives	2
Methods			
Protocol and registration	5	Indicate whether a review protocol exists; state if and where it can be accessed (e.g. a Web address); and if available, provide registration information, including the registration number	1
Eligibility criteria	6	Specify characteristics of the sources of evidence used as eligibility criteria (e.g. years considered, language and publication status), and provide a rationale	3
Information sources	7	Describe all information sources in the search (e.g. databases with dates of coverage and contact with authors to identify additional sources), as well as the date the most recent search was executed	3
Search	8	Present the full electronic search strategy for at least 1 database, including any limits used, such that it could be repeated	Appendix
Selection of sources of evidence	9	State the process for selecting sources of evidence (i.e. screening and eligibility) included in the scoping review	4
Data charting process	10	Describe the methods of charting data from the included sources of evidence (e.g. calibrated forms or forms that have been tested by the team before their use, and whether data charting was done independently or in duplicate) and any processes for obtaining and confirming data from investigators	3
Data items	11	List and define all variables for which data were sought and any assumptions and simplifications made	Table 2
Critical appraisal of individual sources of evidence	12	If done, provide a rationale for conducting a critical appraisal of included sources of evidence; describe the methods used and how this information was used in any data synthesis (if appropriate)	4
Synthesis of results	13	Describe the methods of handling and summarising the data that were charted	4–8
Results			
Selection of sources of evidence	14	Give numbers of sources of evidence screened, assessed for eligibility and included in the review, with reasons for exclusions at each stage, ideally using a flow diagram	Figure 1
Characteristics of sources of evidence	15	For each source of evidence, present characteristics for which data were charted and provide the citations	Table 2
Critical appraisal within sources of evidence	16	If done, present data on critical appraisal of included sources of evidence (see item 12)	4–8
Results of individual sources of evidence	17	For each included source of evidence, present the relevant data that were charted that relate to the review questions and objectives	Table 2
Synthesis of results	18	Summarise and/or present the charting results as they relate to the review questions and objectives	Table 2
Discussion			
Summary of evidence	19	Summarise the main results (including an overview of concepts, themes and types of evidence available), link to the review questions and objectives, and consider the relevance to key groups	8
Limitations	20	Discuss the limitations of the scoping review process	9–10
Conclusions	21	Provide a general interpretation of the results with respect to the review questions and objectives, as well as potential implications and/or next steps	8–9
Funding			
Funding	22	Describe sources of funding for the included sources of evidence, as well as sources of funding for the scoping review. Describe the role of the funders of the scoping review	10

JBI = Joanna Briggs Institute; PRISMA-ScR = Preferred Reporting Items for Systematic Reviews and Meta-Analyses extension for Scoping Reviews.

From: Tricco AC, Lillie E, Zarin W, O'Brien KK, Colquhoun H, Levac D, et al. PRISMA Extension for Scoping Reviews (PRISMA-ScR): Checklist and Explanation. Ann Intern Med. 2018; 169:467–473. doi: 10.7326/M18-0850.

### Stage 1: Identifying the research question

A clear research question was set that would encompass the relevant literature on the topic under study. The question was framed using Population (occupational therapy students), Concept (international collaboration) and Context (online) approach (Peters et al., 2020).

### Stage 2: Identifying relevant studies

All primary research was included. Searching was completed through seven electronic databases (see below) and through hand searching.

### Stage 3: Study selection

Criteria were set post hoc for inclusion into the review. This is to ensure familiarity with the literature prior to setting criteria. The inclusion and exclusion criteria were applied in this stage and studies selected for inclusion into the review.

### Stage 4: Charting the data

The key data was charted from the studies (as in Arksey and O'Malley, 2005) that were included in the review (see Table 3).

### Stage 5: Collating, summarising and reporting the results

The data was then collated to give an overview of the breath of literature (recommended by Levac et al., 2010) with a thematic summary of the main findings.

Two separate database searches (completed by SH and KO) for the review were completed on 2^nd^ January 2020 and again on 3^rd^ September 2020. Two searches were completed to ensure completeness and to allow for new publications that may not have been included in the earlier search. The following databases were searcher for the review: CINAHL, PsycINFO, PubMed, Applied Social Sciences Index and Abstracts, Scopus, British Education Index and OTseeker.

The following search terms were used: ‘occupational therap*’, ‘transnational education’, ‘web-exchange’, ‘internationalisation’, ‘internationalization’, ‘international student collaboration’, ‘internet-based learning’, ‘e-learning’, ‘online learning’, ‘global experiential learning’, ‘international education’, ‘international education’, ‘intercultural learning’, ‘international educational interaction’, ‘synchronous online education’, ‘global partnership’. MESH terms, Boolean logic and truncation were used where appropriate (see OSF link for full detailed search procedure and results: https://mfr.de-1.osf.io/render?url=https://osf.io/vrctu/?direct%26mode=render%26action=download%26mode=render). No search limits were set to ensure the breadth of the literature included all relevant studies on the topic (Arksey & O’Malley, 2005). Key journals and reference lists were also hand searched.

Rayyan, a web application for systematic reviews (Ouzzani et al., 2016) was used throughout the study selection stage process. Duplicates were removed and the screening was completed in three stages using the Rayyan software: 1) the title screen, 2) the abstract and 3) the full-text screen.

The study selection process, deciding on the inclusion and exclusion criteria, was an iterative process, as recommended (Levac et al., 2010). Inclusion criteria for the review are as follows:

- The research describes an online international collaboration.
- The sample included occupational therapy, at any stage of their training.
- The papers were in the English language.
- The papers were primary research including (but not limited to) randomised controlled trials, quasi-experimental design, pilot studies and qualitative studies. Systematic reviews and meta-analysis were also included.

Due to the scoping nature of the review, studies were included regardless of design or quality. Studies were excluded if they were evaluating or describing an interprofessional learning activity, as the outcomes were likely to differ depending on the professions involved. Conference abstracts or posters were excluded as they do not allow for detail of research. Audits, book reviews, opinion pieces, editorials, letters to the editor and study protocols were also excluded. Studies were excluded if the article was retracted or where the full text was not available.

## Results

**Figure 1.**
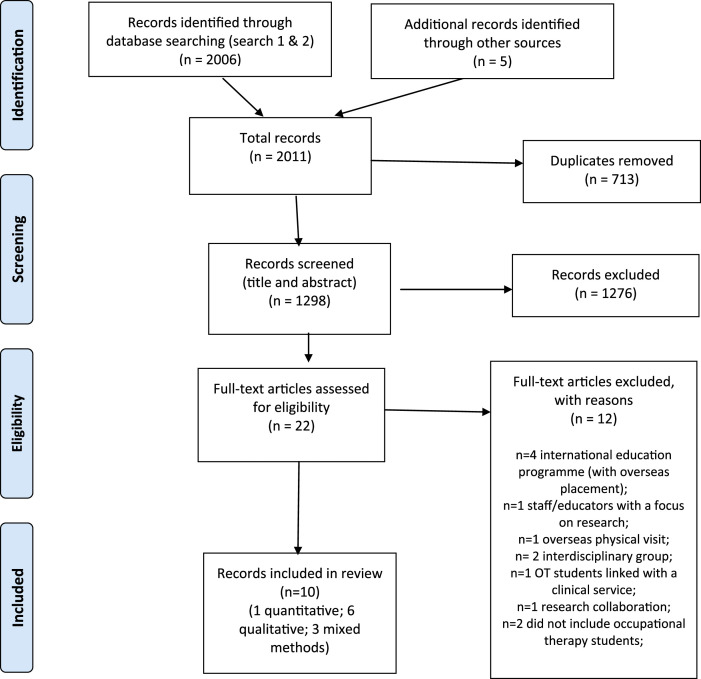
PRISMA 2009 flow diagram.

The database searches yielded 2006 results with an additional five records identified from hand search (see Table 2). Following removal of duplicates and application of inclusion criteria, a total of 22 records were identified for full-text screen, of which ten were included in the full review. A PRISMA flow diagram (Moher et al., 2009) shows each stage of the study selection phase (see Figure 1).

**Table 2.** Results from database search.

Name of database	Papers found search 1	Papers found search 2	Papers rejected after title and abstract screen	Papers accepted for full-text review
CINAHL	216	113	212	5
PUBMED	72	507	366	2
SCOPUS	132	58	91	9
British Education Index	18	0	18	0
Applied Social Sciences Index and Abstracts	238	480	464	3
PsycINFO	15	53	18	1
OTseeker	0	104	104	0
Hand searched from reference lists	5	0	3	2

Following the application of the criteria for inclusion, data from the studies was extracted and then charted. The charting process was continual as studies were included into the review. The charting of included studies can be seen in Table 3. The included studies were critically reviewed, using appropriate CASP form (CASP, 2019), level of evidence ascertained, and following this, the results were summarised thematically and are presented below.

**Table 3.** Charting of included papers.

Author	Aim (for students)	Population	Approach/format	Countries	Frequency and duration	Outcomes	Level of evidence^a^
Aldrich (2015)	To enhance understanding of occupational concepts and global context on same	52 US and 35 Swedish students. Data from US students	Each cohort had preparation prior to online meeting. Student groups of 10–26 students had group interactions (facilitated by faculty) via web conference for 1 month to discuss readings and personal experience on topics	Sweden; US	Four weekly sessions lasting 1 hour	Reflective assignment and course evaluation	Level 6
Aldrich and Grajo (2017)	Help students understand global concerns of occupational therapy and ways to interact	157 US students (BSOS and MOT)	Two different approaches included – mixture of online interactions, reflection and group (online) presentation; social media, email and video conference lectures	US with Sweden; South Africa, Philippines	1–2 synchronous small-group discussion with 4–6 asynchronous discussion	Multicultural Experiences Questionnaire (Narvaez et al., 2009)	Level 6
Aldrich and Johansson (2015)	Connect students to help them understand global context of occupational therapy/science	25 US students and 6 Swedish students	Large student groups of 10–26 students had group interactions (facilitated by faculty) via web conference for 1 month to discuss set topics	Sweden; US	Four weekly sessions lasting 1 hour	Survey and analysis of content of four interactive sessions	Level 6
Aldrich and Peters (2019)	To explore four concepts of occupational justice	21 US and 16 SA	Small group (5–7 students) projects on predetermined readings/topic. Presentation of understanding, application and perceptions of readings by each group with Q&A	South Africa and US	Six video conferences of presentation and Q&A	Questionnaire (adapted from Aldrich and Johansson, 2015)	Level 6
Asher et al. (2014)	To learn about occupational therapy practice in different country	7 US (data reported from 5 US students only)	Web-based video technology used to facilitate discussions. First introduction session focused on occupational therapy practice in each country; second session presented prepared case-studies, planned and discussed goals and intervention plan	US and India	Two sessions (number of sessions and the duration of sessions is not clear)	Student reflection through a survey	Level 6
Cabatan and Grajo (2017)	To enhance intercultural learning and understanding of concepts of occupation	58 Philippines; 102 US	Two different cohorts – different approaches and format taken. A mixture of student virtual conversations (synchronous and asynchronous); faculty presentations; social media, video calls and email used	US and Philippines	At least 1/week (30 mins–1 h); 4 interactions OR at least 30 min (weekly/fortnightly); 3–6 interactions	Reflective essay; survey	Level 6
Psychouli et al. (2020)	To develop students’ cultural competence, specifically the dimension of cultural awareness	32 US; 56 Cyprus	Ten groups of 5 students provided with a topic related to a vulnerable population group. Had 2 weeks to prepare cultural and therapeutic information on group. Private Facebook groups used by students. Virtual exchange took place over 3 days, one hour per group for discussion. Moderator present to prompt students if required	US and Cyprus	One 1-h synchronous video conference (with translation service) per student group	Self-report survey (Cultural Awareness Scale for Occupational Therapy Students; Castro et al., 2017), self-reflection and focus groups	Level 6
Sood et al. (2014)	To help students understand the impact of cultural context on client care	18 US and 13 India	Paediatric assessment and intervention course. Asynchronous communication through online discussion board with assigned reading. Topics related to cultural factors related to children, families, disability and play	India and US	Five sessions over 15 weeks with online discussion of each topic open for 7–10 days	Inventory for Assessing the Process of Cultural Competence among Health Care Professionals–Revised (Campinha-Bacote, 1999) and Self-reflection form (Sood & Cepa, 2010)	Level 6
Wimpenny et al. (2016)	To learn about culturally appropriate and human rights orientated mental health care. To develop problem-solving, leadership, advocacy and team work skills in mental health occupational therapy	215 students (155 UK; 16 Belgium; 29 South Africa)	International discussion forums – Vodcasts from recent graduates on complex case scenario presented on online platform, along with other resources and links. Students explored minimum of two case scenarios over six-week period – reflection, problem-solving and online discussion	UK, South Africa, Belgium	Weekly work in online forum over 6 weeks (duration of weekly sessions not specified)	Interviews with students and learning technologists; weekly reflective narrative; module evaluation; Intercultural Sensitivity Scale Chen and Starosta (2000)	Level 6
Zadnik et al. (2019)	To develop an understanding, appreciation and curiosity of the other culture in order to be able to understand the roles and lives of future clients	Number of students involved not listed	Small-group synchronous discussions focused on topics related to occupational justice relevant to each culture	US and Greece	30 min. One session (unclear) per group	Feedback from debriefing session (very little detail provided)	Unclear as very little detail provided – Level 7
			Content – introduction to the concept of the cultural exchange, creation of a Facebook page for students to introduce themselves, researching topic, participation in the cross-cultural collaboration				

^a^Level of evidence presented using Ackley et al. (2008) and CASP (2019).

### Characteristics of included studies

The ten studies reported on activities that included students in nine different countries. Students from the United States were included in nine studies, South African students in three (Aldrich and Grajo, 2017; Aldrich and Peters, 2019; Wimpenny et al., 2016), Swedish students in two (Aldrich, 2015; Aldrich and Johansson, 2015), Indian students in two (Asher et al., 2014; Sood et al., 2014), with Philippines (Cabatan and Grajo, 2017), Cyprus (Psychouli et al., 2020), Greece (Zadnik et al., 2019) and Belgium and United Kingdom (Wimpenny et al., 2016) reported in one study each.

The studies reported on qualitative data (Aldrich, 2015; Aldrich and Johansson, 2015; Aldrich and Peters, 2019; Cabatan and Grajo, 2017; Zadnik et al., 2019), quantitative data (Aldrich and Grajo, 2017) and a mix of qualitative and quantitative data (Sood et al., 2014). A total of 811 students were included across the ten studies presented (one study did not report on the number of students – Zadnik et al., 2019, and a number of studies only reported on US students – see Table 3). Two of the studies reported on the same type of online collaboration in the same institutions but with different outcomes (Aldrich, 2015; Aldrich and Johansson, 2015). The same cohort of students reported in Aldrich and Grajo (2017) were included in the dataset used in Cabatan and Grajo (2017). The other studies stand alone in the data reported.

The quality of the studies included in the review was low with all ten studies considered to be Level VI evidence or below – evidence from a single descriptive or qualitative study (Ackley et al., 2008). Despite the limitations of the studies, they were included because of the exploratory nature of the review, the limited literature in the area and the emerging nature of these teaching and learning methods in occupational therapy education.

The studies included will be discussed under the following headings: format of the online interactions, reported outcomes of the online interactions, barriers in implementation and facilitators in implementation.

### Format of the online interactions

The activities that were included across the ten studies were video-conferencing (Aldrich, 2015; Aldrich and Grajo, 2017; Aldrich and Johansson, 2015; Aldrich and Peters, 2019; Asher et al., 2014; Psychouli et al., 2020; Zadnik et al., 2019), written reflections (Aldrich and Grajo, 2017; Psychouli et al., 2020), group presentations (Aldrich and Grajo, 2017; Aldrich and Peters, 2019; Psychouli et al., 2020), preparatory reading (Aldrich, 2015; Aldrich and Johansson, 2015; Aldrich and Peters, 2019; Sood et al., 2014; Wimpenny et al., 2016), interactive discussion (Aldrich, 2015; Aldrich and Grajo, 2017; Aldrich and Peters, 2019; Cabatan and Grajo, 2017; Zadnik et al., 2019), introductory session (Zadnik et al., 2019), closed Facebook groups (Psychouli et al., 2020; Zadnik et al., 2019), other social media (Cabatan and Grajo, 2017), emails/virtual conversation (Cabatan and Grajo, 2017), debriefing session (Zadnik et al., 2019), joint online discussion board (Sood et al., 2014; Wimpenny et al., 2016), web-streamed lecture (Cabatan and Grajo, 2017) and recorded lecture (Cabatan and Grajo, 2017).

There was a variation in the aims of the online collaboration in terms of student learning across the studies, as can be seen in Table 3. A range of different approaches were used in the online activities (see Table 3 for detail) but most involved large (Aldrich, 2015; Aldrich and Johansson, 2015; Sood et al., 2014; Wimpenny et al., 2016) or small-group (Aldrich and Grajo, 2017; Aldrich and Peters, 2019; Asher et al., 2014; Cabatan and Grajo, 2017; Psychouli et al., 2020; Zadnik, et al., 2019) interaction with synchronous online interaction.

The variation in the activities undertaken across the different studies makes comparison difficult. For some studies, the activities and format within the activities reported also varied across groups (Aldrich and Grajo, 2017; Cabatan and Grajo, 2017). Aldrich and Grajo (2017) report on results that were collected in different interactions (range 0–2) with three different countries linking with US students in different cohorts. In the papers by Zadnik et al. (2019) and Asher et al. (2014), it is not clear what number of sessions the students participated in. In Aldrich and Peters (2019), it seems that students were not working on presentations with students from the other country, rather they were presenting on the same topic to each other. From what is reported in Sood et al. (2014), it does not appear that the students met online at the same time. The online interaction appeared to be the use of a joint online discussion board where students reflected on the learning on various themes in an asynchronous way. This also appears to be the format that was used by Wimpenny et al. (2016) though further discussion of the format would have clarified this. This would make the interaction (asynchronous only) in these studies very different to that reported in the other included studies.

### Reported outcomes of the online interactions

Six studies focused on increasing cultural competence (Aldrich and Grajo, 2017; Aldrich and Johansson, 2015; Psychouli et al., 2020; Sood et al., 2014; Wimpenny et al., 2016; Zadnik et al., 2019); for three, there was more of an emphasis on understanding occupational concepts and practice within a cultural context (Aldrich, 2015; Aldrich and Peters, 2019; Cabatan and Grajo, 2017), and one study had a broader focus on learning about similarities and difference in occupational therapy practice (Asher et al., 2014). All of the included studies report positive outcomes from the online interactions that they described, though for one study this was only seen in qualitative and not quantitative results (Psychouli et al., 2020).

Aldrich and Grajo (2017) reported that students who had greater number of online international interactions had an increased desire to explore their own prejudices and to become acquainted with those from other backgrounds. There are some mixed results across the cohorts included on the scores on the measure used (Moher et al., 2009) which warrant further discussion. For some students there is no change, for some a decrease and for others an increase. This variation needs to be addressed by the authors and the potential reasons for this discussed considering the aim was to impact positively on critical consciousness related to culture. The variation in the countries that were interacting with the US students, as well as the difference in the approach taken to the interactions would likely influence the results (range from 0 to 2 in number of interactions). The groups that were included were also unbalanced (ranged from 7 to 47 per group) and there was no natural comparison within the groups in the study. The authors used a published measure (the MEQ) to assess change, which was only done in three other studies (Psychouli et al., 2020; Sood et al., 2014; Wimpenny et al., 2016), one of which reported no change in cultural awareness (Psychouli et al., 2020).

Another paper focused on developing students’ understanding of culture (Zadnik et al., 2019), but no reliable data was presented to indicate if this was achieved. There was very little detail provided on important information such as the number of students involved and their demographics. The data that is included is descriptive and no detail of the data collection or analysis process was provided. Similarly, Aldrich (2015) did not include any details of the analysis that was undertaken – it is not clear if any analysis of the data took place at all. Although only data on the US students is included, the author reports that students found the online activities important components of their overall learning in the module within which it was located. Sood et al. (2014) also sought to increase the cultural competence of their students and report that the programme was effective in increasing these perceived levels. Although they used a published measure to capture this data, the groups were too small to carry out the parametric analysis that was undertaken (e.g. n-4; n = 9) and so the results reported are questionable. Psychouli et al. (2020) report no change in cultural awareness following analysis of data. The duration of the interaction, one session per student group, may have been a factor in this. In the qualitative data presented by Sood et al. (2014) there was no description of how the themes were identified in the data. From the reader’s perspective it is not clear if the quotes included were reflective of the data that was gathered. Resultantly, and because a convenience sample was used, the results from this study are over-stated in the paper.

Reflective essays and end-of-programme questionnaires (non-standardised) were used by a number of authors to evaluate outcomes (see Table 3). In the programme described in Cabatan and Grajo (2017) these methods were used and the authors report positive results in terms of students’ ability to deepen their understanding of the links between occupation and culture. Considering the qualitative analysis that was undertaken there is very little depth of discussion in the results and little detail of the analysis undertaken. Aldrich and Peters (2019) also reported improvements in students’ understanding of themselves and others as well as constructs specific to occupational therapy (though no pre-/post-testing took place). There were, however, many challenges that were reported in this study, some of which may be because of students not working together across countries – rather they were presenting to each other on the topics allocated. Having students work together may have reduced the chance of conflict as well as improving the outcomes targeted and having more productive discussions following presentations. Only 40% of all those who participated in the programme completed the questionnaire which may have biased the results presented. There was also a low response rate in the study by Aldrich and Johansson (2015) in particular with the Swedish students (15% response Swedish; 52% US students) and so the positive results reported are skewed towards the experience of the US students. Students perceived the programme as an opportunity to deepen knowledge about occupation and develop cultural competency though the authors also discuss the challenges that presented (discussed below).

### Barriers to implementation

The biggest barrier to implementation, and the most significant because of the impact it had on the interaction, was difficulties with technology (Aldrich and Johansson, 2015; Asher et al., 2014; Cabatan and Grajo, 2017; Zadnik et al., 2019). This includes slow or lost internet connection or audio problems. Other barriers included the following: Facilitators may not be skilled in managing technology (Aldrich and Peters, 2019) and find it challenging when problems arise. Students become frustrated when there is a perceived mismatch of partners (Asher et al., 2014; Cabatan and Grajo, 2017). Students may prefer other types of learning (Cabatan and Grajo, 2017) and find it difficult to engage in the interaction. Student disengagement (Aldrich and Johansson, 2015; Aldrich and Peters, 2019; Wimpenny et al., 2016) with the interaction is a barrier as it can affect the discussions and the experience of other students.

Language can also be a barrier to implementation. If student groups must translate discussion topics for others (Zadnik et al., 2019) this slows the rate of discussion, affects the fluidity, puts added pressure on the ‘translator’ and lengthens the time required for each session. Language – not having fluency can mean that the discussions are superficial (Aldrich and Johansson, 2015). It can affect student confidence (Aldrich and Johansson, 2015) and impact student willingness to get involved in the discussions. Time zone and timetabling issues (Cabatan and Grajo, 2017) can pose a problem and this needs to be well-planned so that no student group is disadvantaged.

### Facilitators in implementation

A good description of the process of course re-design to include online international collaboration is provided by Aldrich (2015). Although the data presented cannot be used as a way to evaluate its’ success given the limitations of the study design, data analysis process and included participants, it provides a clear overview of what is entailed in developing a course like this. Across the included studies, there are methods that appear to help with successful implementation of these collaborations – they include the following:

- If students are motivated to take part (Zadnik et al., 2019) the programme is more likely to succeed. There appears to be certain individual student characteristics (Cabatan and Grajo, 2017), for example, personality and willingness to participate, that is also beneficial to this type of work.
- Use of social media platforms (Cabatan and Grajo, 2017; Psychouli et al., 2020) helps to link students together in an informal way and allows for discussions that go beyond the classroom.
- It can be challenging to carry out these interactions with large groups of students (Psychouli et al., 2020), but in order to maintain motivation, it is important to avoid repetition in the material presented (Aldrich and Peters, 2019).
- Ensuring that the interaction is student-led means that students feel that they have control over the interactions (Aldrich and Johansson, 2015).
- It is important to have someone skilled in group facilitation involved with the students to help with flow of conversation, managing conflict (Aldrich and Peters, 2019).
- Having dedicated course design and technical support, if available, is important for success (Aldrich, 2015; Wimpenny et al., 2016).
- Having adequate time built into the sessions to allow for discussion of topics and account for issues that might arise (Zadnik et al., 2019) such as technological issues is key.
- In order to support the participation of all students it would be recommended to include supports for students who are working in their non-native language (Aldrich and Johansson, 2015) – for example, having translated questions well in advance of the interactive session, or a translator if resources allow (Psychouli et al., 2020).
- In order for students to get the most benefit from the interactions, students should be adequately prepared, considering the novelty of the situation (Aldrich and Johansson, 2015; Zadnik et al., 2019), made aware of their roles and that of the facilitator (Aldrich and Peters, 2019) as well as being familiar with the content of the discussion (Aldrich, 2015). Letting students know that the interaction may be awkward at times (long pauses or conflict in the discussions) may help them being more comfortable in those situations (Aldrich and Johansson, 2015).

### Discussion and implications of the findings

Occupational therapy courses are applying online international collaborations as one method of helping prepare students to explore culture, occupational science and occupational therapy practice. This enables students to think through a new lens (Zadnik et al., 2019). The various international university collaborations and pedagogical approaches delivered confirm that curriculum designers are committed to ensuring their occupational therapy graduates have a global perspective. These collaborations have given groups and whole cohorts of students the opportunity to learn about differences between countries, services, culture and apply occupational science concepts without having to travel abroad (Watkins and Smith, 2018). Online international learning provides the opportunities for all students to participate in an international experience particularly for those that may not be able to complete international fieldwork, study abroad or international service learning due to financial, personal reasons or COVID-19 restrictions (King et al., 2010). This modality also enables international discipline-specific projects, which would be near impossible were it not for online technology (Wimpenny and Orsini-Jones, 2020). As all students in a group or cohort can participate, this pedagogical approach aligns with goals for inclusive education, one that addresses equality, inclusion and diversity but also enables ‘a quality student experience in a digital age’ (IUA, 2018, p. 3).

There have been unprecedented advances in use of digital learning spaces since the beginning of the COVID-19 pandemic. Prior to the pandemic, occupational therapy curricula delivered a range of online international courses using differing formats and technologies. Using technology as a learning space allows for student to learn at a variety of different times, in various locations and using different technologies. This flexibility is often absent in regular curriculum. While some students may value this flexibility, others may not be digitally literate or use social media (Hills et al., 2016; Wimpenny and Orsini-Jones, 2020). Students may also approach technology differently, which will affect their motivation for engagement, this difference is reflected in the visitor and resident theory (White and Le Cornu, 2011). Visitors are those individuals that choose and use a specific technology to complete a specific task (a visitor drops in and out). The continuum progresses to a resident that lives within a network space or spaces (White and Le Cornu, 2011). Students may therefore need specific expectations on participation, training on how to use chosen platforms and a cultural grounding before they begin (Grajo and Aldrich, 2016). The same is true for academic staff who are designing these virtual collaborations.

Known practical challenges in delivering online learning include connectivity, and technology breakdown (Langegard et al., 2021). For the collaboration to run as planned, it is important to have a staff member or technical expert available to support student communication (Grajo and Aldrich, 2016). Additional challenges for international collaborations include timetabling across different universities and time zone differences. Students appear to value learning spaces that offer opportunities for socialisation (asynchronous or synchronous) in small groups facilitating learning through collaborative, participatory pedagogies (Langegard et al., 2021). This review indicated that there is a need for course designers to consider the needs of students in these learning spaces who are working in a non-native language. To date there appear to be better outcomes reported in students who were working in their native language. Where students are using a second language more time should be included in the schedule to allow rest periods and processing time during discussions (Psychouli et al., 2020). Additional supports may also be needed dependent on the student’s ability to converse in the chosen language of the online collaboration. Curriculum designers may need to consider how to, attract, motivate and sustain students in online activities. To maximise student success, students from participating universities as well as their academic staff may be important participants in course design. This may ameliorate the risk of skewing content to one particular language, culture, context, technology or epistemology. These considerations are investments in the future as digital learning is set to transform learning processes in the next decade and part of that digital transformation is to build digital fluency through the removal of structural barriers (IUA, 2018).

In addition to these practicalities, the measurement of the effectiveness of learning activities in relation to learning outcomes is weak due to barriers and facilitators to implementation that appear to be common across the included studies. Educators may need to apply Biggs (2003) model of constructive alignment when planning online international courses to ensure alignment between learning outcomes, student assessments and teaching and learning activities. Assessment modalities or course evaluations may need to be in a form of reliable, validated and appropriate outcome measurement so that the achievement of learning outcomes are evaluated. This will inform best practice going forward.

Online learning will accelerate over the coming years and this review provides an insight into the barriers, challenges, opportunities and prospects for online international learning. This type of learning achieves the goals of the World Federation of Occupational Therapists as well as the goals of many universities for students to experience a broad range of cultural and social development opportunities in a supported digital learning environment (IUA, 2018).

### Limitations of this review

The quality of the research that has taken place in this area to date is low. It would be beneficial to objectively measure the outcomes of these types of collaborations in the areas that they target, namely: cultural competence, civic awareness, professional identity and increase understanding of occupational concepts covered in the curriculum. Further research in this area is therefore indicated.

## Conclusion

This timely scoping review informs those planning to deliver online international curriculum content. Findings provide considerations of constructive alignment, pedagogical approaches and cultural and epistemological considerations. The review indicates that attention is required to the needs of students in an international online learning space to maximise their learning including the use of technology, and the management of students with different languages. Online international learning does appear to meet the overarching goals of the World Federation Minimum Standards for the education of occupational therapy students to be globally connected but specific measurement of attainment of learning goals are indicated.

## Key findings

• Online international learning in occupational therapy has been used to achieve learning outcomes in cultural awareness and understanding of global health issues. It is currently unclear if online international learning achieves this.
• Course content needs to meet the language, cultural and technical issues of an international learning space and have measurable learning outcomes.
• Students need to be prepared, motivated and supported in this novel approach and prefer small group work.

## What the study has added

Online international learning requires careful collaborative planning to maximise student engagement and measurement of learning outcomes. The findings of the review are especially relevant in the current context of online and virtual learning or training due to COVID-19. This review also highlights the need for rigorous research that can provide more definitive answers on the impact of online international learning in occupational therapy education.
